# The biomechanical and histological effects of posterior cruciate ligament rupture on the medial tibial plateau

**DOI:** 10.1186/s13018-017-0551-x

**Published:** 2017-03-23

**Authors:** Zhenhan Deng, Yusheng Li, Zhangyuan Lin, Yong Zhu, Ruibo Zhao

**Affiliations:** Department of Orthopaedics, Xiangya Hospital, Central South University, Changsha, Hunan Province China

**Keywords:** PCL rupture, Medial tibial plateau, Mankin score, MMP-7, TIMP-1

## Abstract

**Background:**

The objective of this study was to investigate the biomechanical and histological effects of the posterior cruciate ligament (PCL) on the medial tibial plateau.

**Methods:**

A total of 12 cadaveric human knee specimens were collected and grouped as follows: the PCL intact group (*n* = 12), the anterolateral bundle rupture group (*n* = 6), the postmedial bundle rupture group (*n* = 6), and the PCL rupture group (*n* = 12). The strain on the anterior, middle, and posterior parts of the medial tibial plateau with an axial loading force at different flexion angles was measured and analyzed, respectively. Forty-eight rabbits were chosen for animal study: surgery was performed on the one side of each rabbit randomly (experimental group), while the other side was taken as control (control group). Every 12 rabbits were culled at each of the four selected time points to collect the medial tibial plateau for morphological and histological observation.

**Results:**

The PCL rupture, either partial or complete, may generate an abnormal load on all the parts of the medial tibial plateau with axial loading at all positions. Noticeable time-dependent degenerative histological changes of the medial tibial plateau were observed in the rabbit models of PCL rupture. Compared with the control group, all the PCL rupture groups exhibited a higher expression of the matrix metalloproteinase-7 (MMP-7) and the tissue inhibitors of metalloproteinase-1 (TIMP-1) at all the time points.

**Conclusions:**

Either partial or complete PCL rupture may generate an abnormal load on all the parts of the medial tibial plateau with axial loading at all the positions and may cause cartilage degeneration on the medial tibial plateau.

## Background

The posterior cruciate ligament (PCL) is the primary restraint to tibial posterior draw, contributing to approximately 90% of the resistance across most of the arc of knee flexion [1]. The strength of PCL is as twice as that of the anterior cruciate ligament (ACL). Although the incidence of PCL injury is relatively lower than that of the ACL injury, it still accounts for 3 to 44% of all knee injuries [2, 3]. Generally speaking, PCL can be classified into two components, namely, the anterolateral bundle (ALB) and the posteromedial bundle (PMB) [4, 5].

The previous studies about PCL injury mainly focus on the post injury changes of knee joint kinematics, the comparison among various PCL reconstruction methods, and the clinical outcomes after surgery [6–9]. Meanwhile, they are mostly concerned about the complete PCL rupture and its effects on the in situ force measurement of other posterior movement that restrict structures and the contact stress of the tibiofemoral joint or patellofemoral joint. The findings about its effects on other structures in the knee joint after PCL injury are very rare [10]. PCL rupture may initiate the compensatory mechanism of other adjacent articular structures in order to maintain the normal functions of the knee, which can lead to degradation and eventually the osteoarthritis (OA) of the knee [11, 12]. OA of the knee is commonly occurred in the medial tibiofemoral joint [13]. As far as we know, the effects of partial PCL rupture on the medial tibial plateau have not been reported yet.

The matrix metalloproteinase-13 (MMP-13) and the tissue inhibitors of metalloproteinase-1 (TIMP-1) are widely recognized biomarkers for cartilage damage and degradation [14, 15]. The studies on the expression level of MMP-13 and TIMP-1 in the medial tibial plateau with a PCL rupture model may help form an insightful understanding of the medial tibial plateau degradation induced by the PCL injury and the pathogenesis of OA [16].

In the present research, specially designed strain gages were placed on the surface of each PCL fiber bundle to monitor the strain changes. The strain on the anterior, middle, and posterior parts of the medial tibial plateau was recorded respectively after partial or complete PCL rupture. Meanwhile, the histological change and the expression of MMP-13 and TIMP-1 in the medial tibial plateau cartilage of rabbits were examined by the hematoxylin and eosin (HE) staining, the toluidine blue staining, and the immunohistochemical methods. The purpose of this study were to investigate the biomechanical effects of partial and complete PCL rupture on the medial tibial plateau, to explore whether PCL rupture could lead to the cartilage degradation of medial tibial plateau, and to understand the biological mechanism for cartilage degeneration.

## Methods

### Subjects

The present study had been approved by the ethics committee of Xiangya Hospital, Central South University (Grant number: 201212062), and was conducted in accordance with the Protocol of Helsinki. A total of 12 cadaveric human knee specimens were collected from the male subjects with an average age of 30.6 years (ranging from 25 to 38 years). The deaths of the subjects were due to accidents or other causes, but the normal structure and function of the knees were kept intact. Informed consent had been obtained from the relatives of all subjects. Macroscopic inspections and radiological examinations were performed to screen fractures, tumors, severe osteoporosis, degenerative joint diseases, and other anomalies out. The posterior drawer test was conducted to rule out the specimens with PCL damage. An approximately 30 cm sample was taken from the joint line for both the femur and tibia. The soft tissues of the proximal portion of the femur were removed, whereas the remaining soft tissues surrounding the knee joint were left intact. A 3-cm longitudinal posterior midline incision was made on the knee joint to expose the PCL. The ends of the femur and tibia were then fixed in cylinders to ensure firm fixation during testing.

### Grouping of partial and complete PCL rupture and test procedure

The mechanical method used in the present study was the same as our previous study [17]. The specimens were grouped as below, following the order in which experiments were conducted: the PCL intact group (*n* = 12), the anterolateral bundle (ALB) rupture group (*n* = 6), the postmedial bundle (PMB) rupture group (*n* = 6), and the PCL rupture group (*n* = 12).

**Fig. 1:**
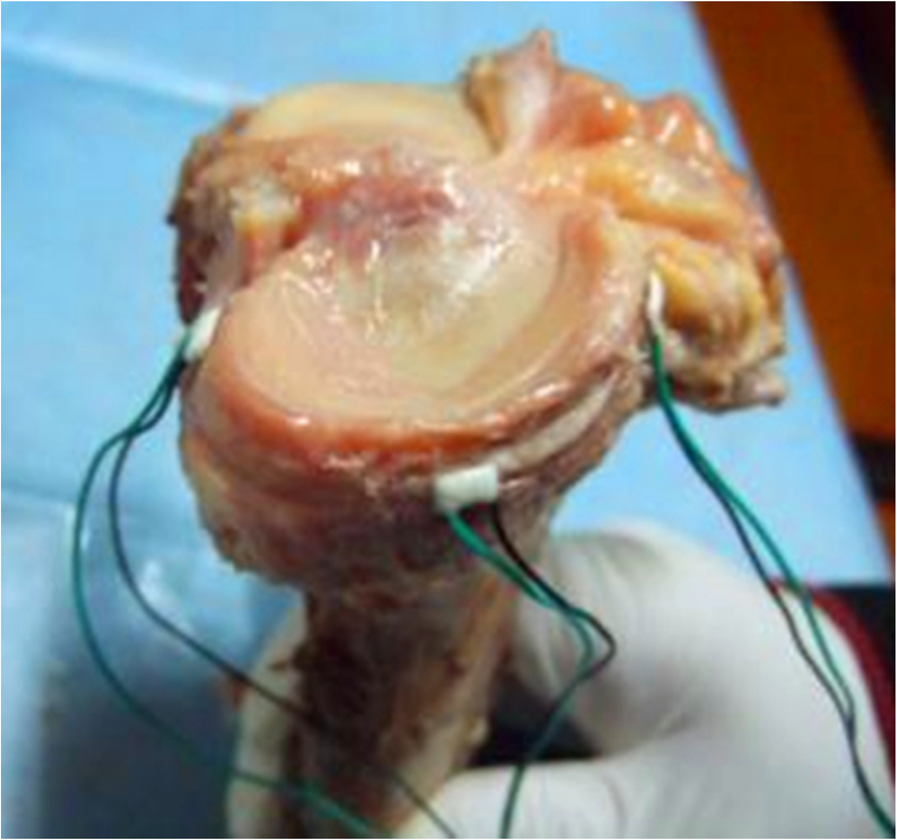
The installment of strain gages at anterior, middle, and posterior part of the medial tibial plateau

The medial parapatellar incision and the medial femoral condyle posterolateral incision were used to expose the anterior, middle, and posterior parts of the medial tibial plateau, and three strain gages were mounted at these parts respectively (Fig. 1). A static strain measuring device was used to measure and record the strain at the aforementioned sites under 200, 400, 600, 800, and 1000 N of loads at 0°, 30°, 60°, and 90° of flexion, respectively. Then, the specimens were randomly divided into the ALB rupture group (*n* = 6) in which the ALB was transected and the PMB rupture group (*n* = 6) in which the PMB was transected. The test procedures were repeated for these two groups. Lastly, the PCL of all the 12 specimens was completely transected to create the PCL rupture group, and the same test procedures were repeated once again.

### PCL rupture animal models

The animal test had been approved by the Medical Ethics Committee of Xiangya Hospital, Central South University (Grant number: 201212067), and was conducted in accordance with the relevant guidelines and regulations. The present study included 48 mature male rabbits (2.5 ± 0.4 kg, 6 months), housed in separated cages at the temperature of 25 °C and the humidity of 50–60% under a 12-h light–12-h dark cycle. All subjects had free access to normal diet and fresh tap water. The experimental animals were raised in the Animal Center of Central South University.

The surgical transection of PCL was performed randomly to one knee, and the contralateral side was exposed without transaction [18]. Briefly, the rabbits were anesthetized by the intraperitoneal administration of 3% sodium pentobarbital (0.03 mg/kg) and fixed on the operation table at the supine position. The drawer test was conducted to examine the stability of both sides of the knee, and a patellar medial incision was used to dissect the joint capsule. Then, the following procedures were executed: (1) put the patella at the lateral dislocation position; (2) expose and transect the PCL at the flexion position of the knee; (3) flush the articular cavity with 3% hydrogen peroxide first and followed by normal saline; and (4) close the incision without fixing the knee joint. The same surgery was operated to the contralateral side without cutting down the PCL. Postoperative anti-infection was performed with intramuscular injection of penicillin (800,000 units) once per day for consecutive 7 days. Any animal with wound infection or suspected infection would be eliminated.

### Morphology and histology

Every 12 rabbits were sacrificed in the 4th, 8th, 16th, and 24th weeks with the medial tibial plateau of both knees being harvested to observe the morphological characteristics, including surface flatness, color, flexibility, and intactness.

The histology method used in the present study was the same as our previous one [19]. Briefly, the paraffin of medial tibial plateau was serial sliced and performed with H&E and immunohistochemical staining. The preprocessed section was then incubated with the 1:300 rabbit polyclonal antibody MMP-13 or TIMP-1 for overnight first, and followed by incubation with the rabbit IgG. The histological changes of the medial meniscus section were observed using a light microscopy and evaluated quantitively by an appropriate scoring system [18, 20]. A Motic Images System was used to evaluate the expression intensity of MMP-13 and TIMP-1 in the specimens. Subsequently, the specimens were observed for cell number correction by light microscopy (at least 10 non-overlapping fields for one side of each rabbit). The results were expressed in a form of positive cell rate (PCR, PCR = positive-staining cell number/total cell number × 100%).

### Statistical analysis

SPSS (version 16.0 for Windows; SPSS Inc., Chicago, IL, USA) was applied for data management and statistical analysis. All the data were expressed as the mean ± SD. The paired *t* test was used to evaluate the paired data. The SNK-q test (Student-Newman-Keuls test) was used to evaluate the pairwise comparison if the mean of data met the homogeneity of variance, while the Dunnett’s T3 test was used instead if the mean of the data did not meet the homogeneity of variance. As for non-parametric test, the Nemenyi rank-sum test and the Wilcoxon rank-sum test were used. A difference with *P* < 0.05 was taken as statistically significant.

## Results

### Strain on the anterior, middle, and posterior parts of the medial tibial plateau under different loads at 0° position in human cadaver testing

At the 0° position of knee flexion, the strain under 200 and 400 N loading force differed significantly neither between the PCL intact group and the ALB rupture group, nor between the PMB rupture group and the PCL rupture group on all the parts (*P* > 0.05, Table 1). The absolute value of strain in the PMB rupture group and the PCL rupture group was significantly larger than that in the PCL intact group and the ALB rupture group on the anterior and middle parts, but smaller on the posterior part (*P* < 0.05). Under the 600, 800, and 1000 N loading force, the difference among the ALB rupture group, the PMB rupture group, and the PCL rupture group was insignificant on all the parts (*P* > 0.05). The absolute value of strain in these three groups was significantly larger than that in the PCL intact group on the anterior and middle parts (*P* < 0.05), but significantly smaller on the posterior part (*P* < 0.05).

**Table 1:** Strain on the anterior, middle, and posterior parts of the medial tibial plateau in all the groups under different loading conditions at different flexion angles during human cadaver testing ($\overline{x}$±s, με)

Flexion angles	Parts	Groups	200 N	400 N	600 N	800 N	1000 N
0°	Anterior	PCL intact group	−17.25 ± 1.71	−33.50 ± 2.24	−48.67 ± 2.90	−60.17 ± 4.15	−78.50 ± 4.01
		ALB rupture group	−18.17 ± 1.94	−34.17 ± 3.43	−66.67 ± 2.88*	−86.33 ± 3.93*	−109.17 ± 4.26*
		PMB rupture group	−25.50 ± 2.43*^,^ **	−45.33 ± 2.66*^,^ **	−65.67 ± 3.14*	−85.00 ± 5.33*	−110.67 ± 3.62*
		PCL rupture group	−26.17 ± 1.40*^,^ **	−45.58 ± 2.91*^,^ **	−68.08 ± 1.98*	−87.17 ± 4.49*	−112.67 ± 4.14*
	Middle	PCL intact group	−12.42 ± 1.44	−21.17 ± 1.85	−32.42 ± 3.06	−43.83 ± 3.54	−54.75 ± 4.27
		ALB rupture group	−12.83 ± 1.72	−21.83 ± 2.14	−42.83 ± 3.19*	−59.50 ± 3.73*	−73.50 ± 3.94*
		PMB rupture group	−17.00 ± 1.90*^,^ **	−27.83 ± 1.72*^,^ **	−42.33 ± 3.56*	−59.17 ± 4.71*	−73.83 ± 3.25*
		PCL rupture group	−17.33 ± 1.72*^,^ **	−28.25 ± 2.30*^,^ **	−44.08 ± 3.32*	−61.50 ± 4.58*	−75.67 ± 4.54*
	Posterior	PCL intact group	−6.17 ± 1.34	−10.50 ± 2.11	−15.17 ± 1.90	−20.33 ± 3.03	−25.50 ± 3.34
		ALB rupture group	−5.50 ± 1.05	−9.67 ± 1.63	−7.83 ± 2.79*	−9.50 ± 3.56*	−13.67 ± 3.72*
		PMB rupture group	−2.83 ± 2.23*^,^ **	−4.83 ± 1.84*^,^ **	−7.17 ± 2.14*	−9.0 ± 3.16*	−12.33 ± 3.50*
		PCL rupture group	−2.42 ± 1.88*^,^ **	−4.67 ± 2.02*^,^ **	−6.17 ± 3.41*	−9.33 ± 3.06*	−11.33 ± 3.23*
30°	Anterior	PCL intact group	−5.33 ± 2.15	−10.42 ± 2.39	−15.50 ± 3.18	−21.08 ± 3.68	−26.75 ± 4.96
		ALB rupture group	−5.83 ± 1.17	−11.50 ± 3.39	−36.17 ± 3.55*	−47.67 ± 4.18*	−60.00 ± 4.56*
		PMB rupture group	−5.67 ± 1.97	−10.83 ± 2.56	−17.33 ± 3.83**	−21.67 ± 4.80**	−28.83 ± 5.88**
		PCL rupture group	−14.08 ± 1.73*^,^ **^,^ ***	−25.50 ± 3.03*^,^ **^,^ ***	−38.42 ± 3.42*^,^ ***	−49.00 ± 4.51*^,^ ***	−61.75 ± 5.15*^,^ ***
	Middle	PCL intact group	−23.25 ± 2.01	−39.33 ± 3.11^a^	−57.92 ± 4.94	−77.83 ± 3.13	−97.08 ± 4.68
		ALB rupture group	−23.83 ± 2.14	−41.17 ± 3.87^a^	−82.33 ± 4.80*	−104.83 ± 4.26*	−126.83 ± 5.08*
		PMB rupture group	−23.33 ± 1.97	−40.17 ± 3.19^a^	−59.67 ± 5.61**	−79.00 ± 4.94**	−98.50 ± 5.47**
		PCL rupture group	−31.17 ± 2.41*^,^ **^,^ ***	−56.58 ± 2.47*^,^ **^,^ ***^, a^	−84.25 ± 4.58*^,^ ***	−108.50 ± 4.54*^,^ ***	−127.83 ± 5.65*^,^ ***
	Posterior	PCL intact group	−12.50 ± 2.24	−23.00 ± 3.33	−34.25 ± 3.42	−41.67 ± 4.60	−48.33 ± 4.48
		ALB rupture group	−11.00 ± 2.37	−20.67 ± 3.08	−19.83 ± 3.55*	−23.50 ± 4.09*	−29.67 ± 5.85*
		PMB rupture group	−11.83 ± 2.14	−20.67 ± 3.14	−33.50 ± 3.62**	−40.00 ± 4.56**	−46.50 ± 5.82**
		PCL rupture group	−6.67 ± 2.02*^,^ **^,^ ***	−12.58 ± 2.75*^,^ **^,^ ***	−17.83 ± 3.56*^,^ ***	−22.58 ± 3.20*^,^ ***	−27.33 ± 5.05*^,^ ***
60°	Anterior	PCL intact group	8.75 ± 1.77	17.50 ± 2.88	25.00 ± 3.67	31.75 ± 4.16	37.17 ± 4.06
		ALB rupture group	7.33 ± 1.97	16.50 ± 2.51	10.83 ± 3.82*	15.00 ± 4.94*	17.83 ± 4.83*
		PMB rupture group	8.00 ± 1.41	16.83 ± 2.48	23.50 ± 3.62**	31.00 ± 3.90**	36.50 ± 3.56**
		PCL rupture group	3.33 ± 1.50*^,^ **^,^ ***	5.92 ± 2.31*^,^ **^,^ ***	8.83 ± 3.16*^,^ ***	13.67 ± 4.25*^,^ ***	16.42 ± 4.32*^,^ ***
	Middle	PCL intact group	−25.50 ± 2.39	−43.00 ± 3.02	−64.83 ± 3.46	−83.50 ± 5.28^a^	−108.67 ± 5.40
		ALB rupture group	−26.00 ± 2.28	−44.50 ± 2.81	−86.17 ± 3.60*	−114.33 ± 5.61*^, a^	−141.50 ± 5.72*
		PMB rupture group	−25.67 ± 2.34	−43.67 ± 2.25	−66.50 ± 5.21**	−85.83 ± 6.91**^, a^	−110.00 ± 5.40**
		PCL rupture group	−34.75 ± 1.87*^,^ **^,^ ***	−59.33 ± 3.09*^,^ **^,^ ***	−87.58 ± 3.50*^,^ ***	−116.33 ± 4.01*^,^ ***^, a^	−144.08 ± 5.37*^,^ ***
	Posterior	PCL intact group	−24.75 ± 3.11	−41.58 ± 5.09	−62.17 ± 6.09	−82.92 ± 8.04^a^	−105.42 ± 6.65
		ALB rupture group	−23.00 ± 3.41	−39.33 ± 5.47	−34.83 ± 7.25*	−41.33 ± 5.20*^, a^	−48.17 ± 10.76*
		PMB rupture group	−23.67 ± 3.08	−40.50 ± 4.59	−60.50 ± 5.09**	−79.67 ± 5.20**^, a^	−103.50 ± 9.79**
		PCL rupture group	−11.75 ± 4.03*^,^ **^,^ ***	−23.33 ± 3.99*^,^ **^,^ ***	−33.17 ± 6.97*^,^ ***	−40.08 ± 5.99*^,^ ***^, a^	−46.42 ± 9.32*^,^ ***
90°	Anterior	PCL intact group	11.33 ± 2.46	20.67 ± 4.25^a^	30.00 ± 3.30^a^	38.25 ± 4.16	46.17 ± 3.46
		ALB rupture group	9.50 ± 2.51	19.00 ± 2.97^a^	12.50 ± 3.94*^, a^	18.33 ± 3.01*	23.50 ± 5.21*
		PMB rupture group	11.00 ± 1.55	20.00 ± 3.41^a^	28.50 ± 3.94**^, a^	37.83 ± 3.60**	44.17 ± 3.66**
		PCL rupture group	4.42 ± 1.51*^,^ **^,^ ***	8.50 ± 2.94*^,^ **^,^ ***^, a^	11.33 ± 2.46*^,^ ***^, a^	17.58 ± 4.44*^,^ ***	20.75 ± 4.14*^,^ ***
	Middle	PCL intact group	−23.25 ± 2.77	−41.42 ± 3.32	−60.92 ± 4.32	−85.58 ± 4.25	−102.42 ± 6.74
		ALB rupture group	−25.17 ± 3.25	−42.67 ± 3.45	−84.83 ± 5.08*	−115.17 ± 4.88*	−146.67 ± 7.97*
		PMB rupture group	−24.50 ± 2.59	−42.17 ± 3.19	−61.67 ± 4.59**	−87.50 ± 5.47**	−104.33 ± 8.80**
		PCL rupture group	−34.25 ± 2.34*^,^ **^,^ ***	−57.58 ± 2.23*^,^ **^,^ ***	−86.33 ± 4.31*^,^ ***	−116.42 ± 4.50*^,^ ***	−148.67 ± 6.36*^,^ ***
	Posterior	PCL intact group	−36.58 ± 3.75	−64.83 ± 4.39	−95.42 ± 5.87	−125.33 ± 4.83	−153.67 ± 7.58^a^
		ALB rupture group	−35.17 ± 3.19	−62.00 ± 4.60	−69.50 ± 6.86*	−91.67 ± 4.68*	−116.67 ± 11.99*^, a^
		PMB rupture group	−34.00 ± 3.41	−61.83 ± 4.62	−92.83 ± 5.98**	−123.17 ± 4.02**	−150.67 ± 8.50**^, a^
		PCL rupture group	−25.58 ± 2.94*^,^ **^,^ ***	−44.00 ± 3.72*^,^ **^,^ ***	−66.50 ± 6.90*^,^ ***	−90.92 ± 5.27*^,^ ***	−112.25 ± 8.34*^,^ ***^, a^

*PCL* posterior cruciate ligament, *ALB* anterolateral band, *PMB* posteromedial band

*P < 0.05 compared with PCL intact; **P < 0.05 compared with ALB rupture; ***P < 0.05 compared with PMB rupture

^a^Dunnett T3 test; others, SNK-q test

### Strain on the anterior, middle, and posterior parts of the medial tibial plateau under different loads at 30° flexion in human cadaver testing

At the 30° flexion, the difference among the PCL intact group, the ALB rupture group, and the PMB rupture group under 200 and 400 N loading force was not significantly different on all the parts (*P* > 0.05, Table 1). The absolute value of strain in these three groups was significantly smaller than that in the PCL rupture group on the anterior and middle part (*P* < 0.05), but significantly larger on the posterior part (*P* < 0.05). Under the 600, 800, and 1000 N loading force, the strain differed significantly neither between the PCL intact group and the PMB rupture group, nor between the ALB rupture group and the PCL rupture group on all the parts (*P* > 0.05). The absolute value of strain in the ALB rupture group and the PCL rupture group was significantly larger than that in the PCL intact group and the PMB rupture group on the anterior and middle parts (*P* < 0.05), but significantly smaller on the posterior part (*P* < 0.05).

### Strain on the anterior, middle, and posterior part of the medial tibial plateau under different loads at 60° and 90° flexion in human cadaver testing

At the 60° and 90° flexion, the difference among the PCL intact group, the ALB rupture group, and the PMB rupture group under 200 and 400 N loading force was insignificant on all the parts (*P* > 0.05, Table 1). The absolute value of strain in these three groups was significantly larger than that in the PCL rupture group on the anterior and posterior part (*P* < 0.05), but significantly smaller on the middle part (*P* < 0.05). Under the 600, 800, and 1000 N loading force, the strain differed significantly neither between the PCL intact group and the PMB rupture group, nor between the ALB rupture group and the PCL rupture group on all the parts (*P* > 0.05). The absolute value of strain in the ALB rupture group and the PCL rupture group was significantly smaller than that in the PCL intact group and the PMB rupture group on the anterior and posterior part (*P* < 0.05), but significantly larger on the middle part (*P* < 0.05).

### Morphology and histology of medial tibial plateau in the PCL rupture rabbit model

**Fig. 2:**
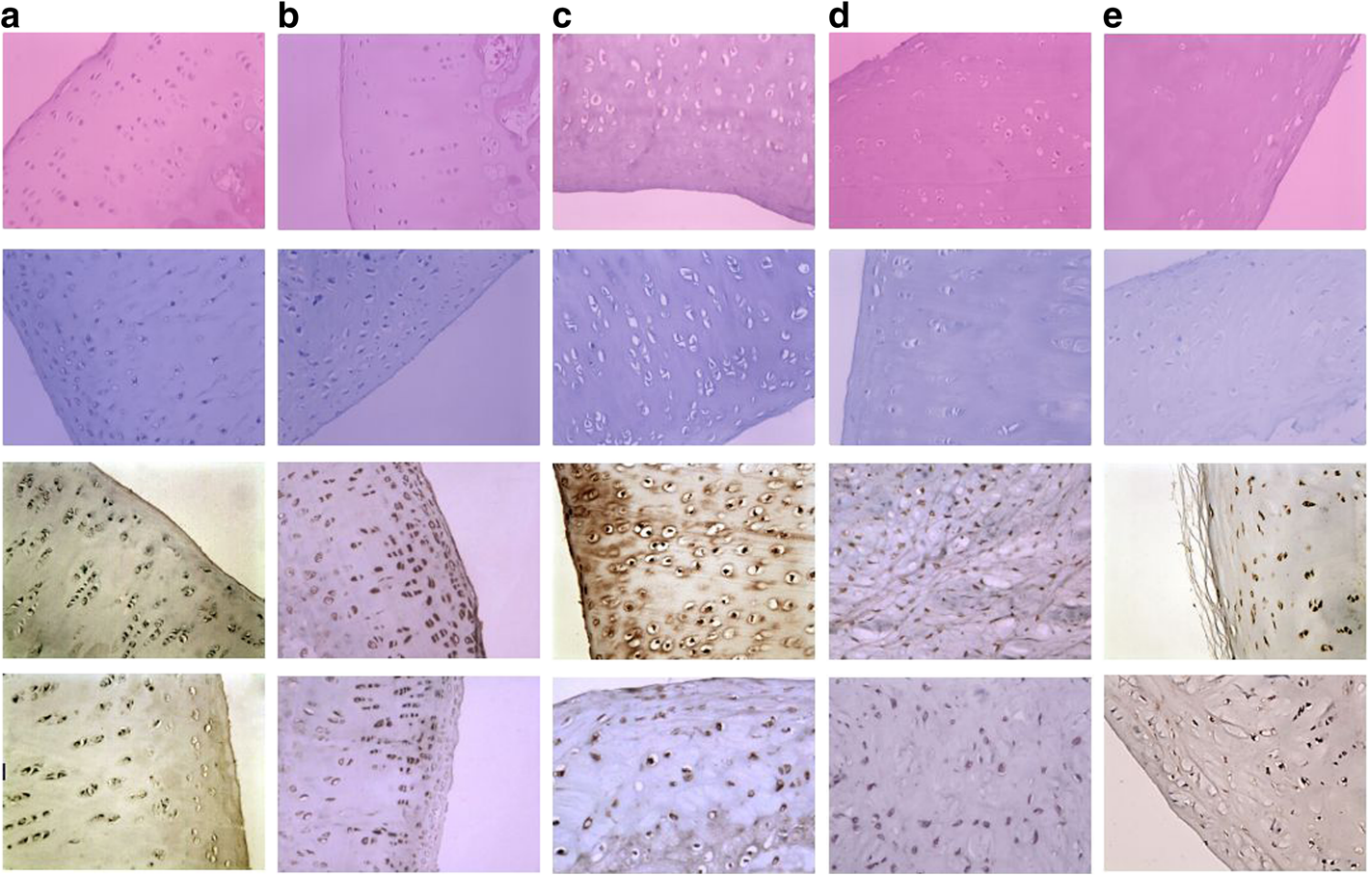
The *first* and *second line* images presented the histological characteristics of the medial tibial plateau by H&E staining and toluidine *blue* staining, respectively. The *third* and *fourth line* images presented the MMP-13 and TIMP-1 expression in the PCL rupture side and the control side, respectively. **a** The control side: the surface was continuous and smooth, chondrocytes in normal shape and regularly ranged, basicytes capsule surrounded the cells, clear and dense tissue layers, long axis of the cell parallel to the joint surface, shape were similar to fiber cells. The cells in the middle and deep layers were round, continuous tidemark, no vessel hyperplasia and inflammatory cell formation; even *dark blue* in toluidine *blue* staining; barely MMP-13 and TIMP-1 positive-staining cells were detected in chondrocytes. **b** Fourth week after PCL rupture: smooth and approximately flat surface, superficial layer cells are in *spindle* shape, long axis of the cell parallel to the joint surface, tissue layers and transitional layer cells were slightly disorderly arranged, no clustering of hypertrophy cells, continuous tidemark; even toluidine blue staining; limited number of MMP-13 and TIMP-1 positive-staining cells were detected. **c** Eighth week after PCL rupture: slight rough surface, collagen fibers were unevenly thick and arranged disorderly, chondrocytes were *round* in shape, occasionally fissure and cartilage defects, disorderly arranged cells and tissue layers, common clusters, cell hypertrophy and hyperplasia, vague tidemarks but still continuous; uneven toluidine blue staining, superficial layer light staining; more MMP-13 and TIMP-1 positive-staining cells were observed in the superficial layer and partial middle layer. **d** Sixteenth week after PCL rupture: extremely rough surface, common depression and deep fissures into the middle layer, superficial and partial radial layer fibrosis, loose tissue and disorderly arrangement of tissue layers, decreased cell numbers and uneven distribution, gathered in clusters, discontinuous tidemarks, occasionally slightly pannus hyperplasia; extremely uneven toluidine *blue* staining, partial loss of dye in deep cartilage layer; MMP-13 and TIMP-1 positive-staining cells were scattered in cytoplasm of full layer. **e** Twenty-fourth week after PCL rupture: fracture surface, deep fissures into the radial layer, cartilage thickness and cell number decreased obviously, layers disorderly arranged, large range of cartilage fibrosis, loose tissue, tidemarks disappeared, wide-scope or full-layer loss dye of toluidine blue staining; MMP-13 and TIMP-1 positive-staining cells decreased and located only in superficial layers

Compared with the control groups, the cartilage of medial tibial plateau in the PCL rupture group exhibited obvious degenerative characteristics (Table 2). The HE and toluidine blue staining histology of medial tibial plateau in the PCL rupture groups showed time-dependent abnormalities and deterioration in comparison with the control groups, indicating that PCL rupture may act as a progressive degenerative factor for the medial tibial plateau (Fig. 2).

**Table 2:** Morphological characteristics of the medial tibial plateau between the control groups and the PCL rupture groups in the PCL rupture rabbit model

	Control group	PCL rupture group			
	All time points	4th week	8th week	16th week	24th week
Structural integrity	Integrated	Integrated	Integrated	Worn free edge	Avulsion
Surface	Smooth	Smooth	Not smooth	Rough	Rough
Color	Bright white	Light blue	Faint yellow	Gray-yellow	Gray-yellow
Elasticity	Good	Good	Slight slack	Slack	Slack

### Mankin score, MMP-13 and TIMP-1 expression in the medial tibial plateau of PCL rupture rabbit model

The Mankin score, the MMP-13 and TIMP-1 expression level in the cartilage of the medial tibial plateau of the control and the PCL rupture groups at all the time points were presented in Table 3. The pairwise comparisons for the Mankin score, the MMP-13 and TIMP-1 expression level among all the time points were presented in Table 4. In the 4th week after PCL rupture, no significant difference in the Mankin score of the medial tibial plateau cartilage was observed between the control group and the PCL rupture groups (*P* > 0.05). Since the 8th week after PCL rupture, the Mankin score of the medial tibial plateau cartilage in the PCL rupture groups became significantly higher compared to the control group (*P* < 0.05). Meanwhile, the Mankin score of the PCL rupture groups increased continuously from the 4th week to the 24th week after operation, and the difference was significant (*P* < 0.05). On the contrary, the change of the Mankin score was insignificant in the control group (*P* > 0.05).

**Table 3:** Comparison of Mankin score, the MMP-13 and TIMP-1 expression level in the medial tibial plateau at all the time points between the PCL rupture groups and the control groups in the PCL rupture rabbit model (Wilcoxon rank-sum test)

		4th week	8th week	16th week	24th week
Mankin score	PCL rupture group	0.58 ± 0.67	5.67 ± 0.89	8.33 ± 0.89	11.00 ± 1.28
	Control group	0.42 ± 0.52	0.42 ± 0.67	0.50 ± 0.67	0.58 ± 0.67
	*P* value	>0.05	<0.05	<0.05	<0.05
MMP-13	12.95 ± 1.55	50.95 ± 4.30	43.87 ± 4.14	17.02 ± 0.90	12.95 ± 1.55
	4.11 ± 2.59	4.70 ± 3.36	4.46 ± 3.00	4.77 ± 2.37	4.11 ± 2.59
	*P* value	<0.05	<0.05	<0.05	<0.05
TIMP-1	PCL rupture group	10.03 ± 0.89	31.86 ± 3.77	29.85 ± 3.57	5.99 ± 0.98
	Control group	4.95 ± 3.26	5.55 ± 3.82	5.13 ± 3.56	4.67 ± 2.90
	*P* value	<0.05	<0.05	<0.05	>0.05

**Table 4:** Pairwise comparison of Mankin score, the MMP-13 and TIMP-1 expression level in the medial tibial plateau at various time points between the PCL rupture groups and the control groups in the PCL rupture rabbit model (*P* value of Dunnett T3 test)

		4 W:8 W	4 W:16 W	4 W:24 W	8 W:16 W	8 W:24 W	16 W:24 W
Mankin score	PCL rupture group	<0.05	<0.05	<0.05	<0.05	<0.05	<0.05
	Control group	>0.05	>0.05	>0.05	>0.05	>0.05	>0.05
MMP-13	PCL rupture group	<0.05	<0.05	<0.05	<0.05	<0.05	<0.05
	Control group	>0.05	>0.05	>0.05	>0.05	>0.05	>0.05
TIMP-1	PCL rupture group	<0.05	<0.05	<0.05	>0.05	<0.05	<0.05
	Control group	>0.05	>0.05	>0.05	>0.05	>0.05	>0.05

The MMP-13 expression in all the PCL rupture groups was higher compared to the control group (*P* < 0.05, Fig. 2). In the PCL rupture groups, the expression of MMP-13 was lower in the 4th week compared to that in the 8th, 16th, and 24th weeks (*P* < 0.05), and higher in the 8th week compared to that in the 4th, 16th, and 24th weeks (*P* < 0.05). There were no significant differences among the control groups (*P* > 0.05).

In the 4th, 8th, and 16th weeks, the TIMP-1 expression in the PCL rupture groups was higher compared to the control groups (*P* < 0.05, Fig. 2), while in the 24th week, there was no significant difference between the PCL rupture groups and the control group (*P* > 0.05). The TIMP-1 expression in the PCL rupture groups was lower in the 24th week compared to that in the 4th, 8th, and 16th weeks (*P* < 0.05), and there was no significant difference between the 8th week and the 16th week (*P* > 0.05). The TIMP-1 expression was higher in the 8th and 16th weeks compared to that in the 4th and 24th weeks (*P* < 0.05). There were no significant differences among the control groups (*P* > 0.05).

## Discussion

To the best of our knowledge, this was the first study that examined the effects of partial PCL rupture on the medial tibial plateau. The strain on the anterior, middle, and posterior part of the medial tibial plateau was measured after partial and complete PCL rupture for five loading conditions and four flexion angles. When PCL is intact, the strain on all the parts of the medial tibial plateau was compressive and could be ranked in the descending sequence as follows: the anterior, the middle, and the posterior part. Then, the strain on each part increased with the increase of the axial loading. The incomplete linear relationship between the strain and load may be explained by the viscoelasticity and the multiple stresses born by the medial tibial plateau born; meanwhile, the contact point of the tibiofemoral joint retruded gradually as the flexion angle increased. At the 60° position, with the retrution of the tibiofemoral joint contact point and the change of curvature radius of the medial femoral condyle, the anterior part no longer contacted with the medial femoral condyle. The compressive strain therefore turned into tensile strain due to the traction from joint capsule and transverse ligament. The contact point of the tibiofemoral joint was located between the middle and the posterior part, so the strain here was higher. At the 90° of flexion, the tensile strain on the anterior part increased, while the strain on the middle part slightly decreased; and mostly concentrated on the posterior part. When PCL was transected, the strain on the knee joint would be redistributed due to the transference of the PCL stress and the change of the tibiofemoral joint kinematics. The strain on all the parts changed significantly for different flexion angles and loads. At the 0° and 30° position, due to the forward shift of the contact point of the tibiofemoral joint caused by tibial retrusion, the strain on the anterior and middle part increased significantly, while the strain on the posterior part decreased. At the 60° and 90° position, due to the forward shift of the contact point of the tibiofemoral joint again, the tensile strain on the anterior part decreased, and the compressive strain on the posterior part continued decreasing, while the strain on the middle part increased significantly.

Under a relatively smaller load (200 and 400 N), the difference between the PCL intact group and the ALB rupture group was insignificant for the various sites and flexion angles. The tension of PMB might remain at a certain level in the flexion range from 0° to 90°. PMB could still maintain the posterior stability of the tibia even in the condition of ALB rupture. However, the effects on the medial tibial plateau differed in the PMB rupture group: at the 0° position, no difference was observed between the PMB rupture group and the PCL rupture group. This is probably because that ALB is at a low tension state at this position, and the wavy crimpable portion of the fibrillar collagen is geting stretched. Therefore, the ligament is extended without apparent increase of loads. In addition, the tibia retruded slightly at the extension position after PCL rupture. Therefore, the low tensive ALB could not restrain the tibia retrusion after PMB rupture within a certain range. From 30° to 90°, the tension of ALB increased significantly and became higher than that of PMB; as a result, it attained the ability to restrain tibia retrusion after PMB rupture. No statistic difference was observed between the PMB rupture group and the PCL intact group. Under a relatively larger load (600, 800, and 1000 N), PMB could still retain a certain tensity at all the positions. The cross-sectional area of ALB is twice as large as that of PMB [21]. Some scholars even supposed that the posterior fibers only took up 15% of the whole PCL volume [22]. Probably because of the relatively small size of PMB on the PCL section, a small portion of fiber bundles are slacked or damaged due to strain concentration. As a result, there was no significant difference between the ALB rupture group and the PCL rupture group. The results differed in the PMB rupture group for all the angles: at the 0° position, the strain among the PMB rupture group, the ALB rupture group, and the PCL rupture group was not significantly different. This is probably due to the low tension state of ALB at this moment and the relatively small cross-sectional area of PMB. From 30° to 90°, no difference was observed between the PMB rupture group and the intact group, but there were differences among the PMB rupture group, the ALB rupture group, and the PCL rupture group. Probably, due to the drastic increase of the strain of ALB and its wide cross-sectional area, ALB has been able to restrain the tibia retrusion. Therefore, PMB plays the role to maintain stability at all flexion angles under a low load. The strain changes insignificantly at all the parts of the medial tibial plateau after ALB rupture, which provides a theoretical foundation for isometrical reconstruction. ALB exerts the function of maintaining posterior stability from 30° to 90°. The strain changes insignificantly at all the parts of the medial tibial plateau after PMB rupture, which provides a theoretical foundation for double-bundle reconstruction.

Furthermore, based on the previous research findings about the articular cartilage degeneration secondary to PCL rupture in rabbit knees, the present study also examined the morphology and histology changes of the medial tibial plateau after PCL rupture, in order to evaluate its degeneration [23]. The 4th, 8th, 16th, and 24th weeks after PCL rupture were targeted as the time points for observation. In accordance with Wang’s results, this study identified a time-dependent characteristic of the medial tibial plateau degeneration along with the time progression after PCL rupture: no obvious change of cartilage was observed in the 4th week after PCL rupture, but a higher expression of MMP-13 and TIMP was detected in the experimental group, suggesting that they are both sensitive markers for cartilage degradation. In the 8th week, the macroscopic and microscopic mild degradations were observed, which indicated an early stage of OA. The MMP-13 expression level reached the peak value. The previous study also demonstrated a significant elevation of the MMP-13 expression at the early stage of OA in rabbit models, and observed that the MMP-13 expression was mainly distributed at the superficial level of cartilage [24, 25]. Meanwhile, TIMP-1 also exhibited an obviously higher expression compared with that in the 4th week after surgery. It suggested that both catabolism and anabolism in the cartilage matrix were enhanced at the early stage of OA, and the former might be the dominant effect as the level of MMP-13 had a more significant elevation compared with TIMP-1. Moderate cartilage degradation emerged in the 16th week, but presented a weaker expression of MMP-13 compared with the previous time point. This might be due to its regulatory mechanism, though the reason was not certain yet. There was no significant difference in the TIMP-1 expression between the 8th week and 16th weeks. This was consistent with the findings of Bluteau who reported that TIMP-1 remained at a stable level after the early stage of elevation in an ACL rupture model of rabbit [24]. We infer the repair mechanism of cartilage maintained at relative active level at early and intermediate stage. In the 24th week, the wide range of fibrosis, extremely reduced cell number, and disordered ranged tissue layers were evidence of severe cartilage degradation. The significant decrease in the MMP-13 and TIMP-1 expression might be caused by the cell reduction and anabolism obstruction of chondrocytes. The expression of TIMP-1 almost reached its initial stage and exhibited no difference compared with the control group, indicating a catabolism state as the repair function of cartilage exceeded its compensation limit. In the control group, the expression level of MMP-13 and TIMP-1 almost remained unchanged during the experiment, indicating a balance between these two substances in normal cartilage. MMP-13 showed a high expression at all the time points, while TIPM-1 showed a high expression at the early and medial stage of OA. The expression level of MMP-13 was elevated more obviously than that of TIMP-1. It indicated that the MMP-13-induced catabolism played a key role in causing the imbalance between anabolism and catabolism of cartilage and resulting in severe cartilage damage.

One of the limitations of this study lied in the relatively small sample size. More definitive conclusions may be drawn if larger-scale investigations can be carried out. Besides, rabbit knees instead of human knees were used for histological analysis in the present study. Though the rabbit knee might be biomechanically different from that of human’s for its higher degree of flexion and gait, it is similar in gross appearance to that of the human’s except for a smaller patella relative to other structures. Therefore, it was regarded as a good model to simulate the human PCL rupture [26].

## Conclusions

In consideration of the biomechanical property, the anatomy of ligament insertions, and the cluster analysis results, PCL may be classified into the anterolateral functional fiber bundle and the posteromedial functional fiber bundle. PCL rupture may cause an abnormal load on all parts of the medial tibial plateau with any kind of axial loading on all the positions, and may cause cartilage degeneration on the medial tibial plateau. The increased expression level of MMP-13 and TIMP-1 suggests that they may be involved in the process of cartilage degeneration of the medial tibial plateau induced by PCL rupture.

## Abbreviations

ACL: Anterior cruciate ligament
ALB: Anterolateral bundle
HE: Hematoxylin and eosin
MMP-7: Matrix metalloproteinase-7
OA: Osteoarthritis
PCL: Posterior cruciate ligament rupture
PCR: Positive cell rate
PMB: Posteromedial bundle
SNK-q test: Student-Newman-Keuls test
TIMP-1: Tissue inhibitors of metalloproteinase-1

## References

[1] Amis AA, Gupte CM, Bull AM, Edwards A (2006). Anatomy of the posterior cruciate ligament and the meniscofemoral ligaments. Knee Surg Sports Traumatol Arthrosc.

[2] Harner CD, Xerogeanes JW, Livesay GA, Carlin GJ, Smith BA, Kusayama T (1995). The human posterior cruciate ligament complex: an interdisciplinary study. Ligament morphology and biomechanical evaluation. Am J Sports Med.

[3] Chandrasekaran S, Ma D, Scarvell JM, Woods KR, Smith PN (2012). A review of the anatomical, biomechanical and kinematic findings of posterior cruciate ligament injury with respect to non-operative management. Knee.

[4] Race A, Amis AA (1994). The mechanical properties of the two bundles of the human posterior cruciate ligament. J Biomech.

[5] Voos JE, Mauro CS, Wente T, Warren RF, Wickiewicz TL (2012). Posterior cruciate ligament: anatomy, biomechanics, and outcomes. Am J Sports Med.

[6] Mutnal A, Leo BM, Vargas L, Colbrunn RW, Butler RS, Uribe JW (2015). Biomechanical analysis of posterior cruciate ligament reconstruction with aperture femoral fixation. Orthopedics.

[7] Kim SJ, Jung M, Moon HK, Kim SG, Chun YM (2011). Anterolateral transtibial posterior cruciate ligament reconstruction combined with anatomical reconstruction of posterolateral corner insufficiency: comparison of single-bundle versus double-bundle posterior cruciate ligament reconstruction over a 2- to 6-year follow-up. Am J Sports Med.

[8] Jain V, Goyal A, Mohindra M, Kumar R, Joshi D, Chaudhary D (2016). A comparative analysis of arthroscopic double-bundle versus single-bundle posterior cruciate ligament reconstruction using hamstring tendon autograft. Arch Orthop Trauma Surg.

[9] Yoon KH, Bae DK, Song SJ, Cho HJ, Lee JH (2011). A prospective randomized study comparing arthroscopic single-bundle and double-bundle posterior cruciate ligament reconstructions preserving remnant fibers. Am J Sports Med.

[10] Lei P, Sun R, Hu Y, Li K, Liao Z (2015). Effect of posterior cruciate ligament rupture on the radial displacement of lateral meniscus. Clin Biomech (Bristol, Avon).

[11] Masouros SD, McDermott ID, Amis AA, Bull AM (2008). Biomechanics of the meniscus-meniscal ligament construct of the knee. Knee Surg Sports Traumatol Arthrosc.

[12] Aroen A, Sivertsen EA, Owesen C, Engebretsen L, Granan LP (2013). An isolated rupture of the posterior cruciate ligament results in reduced preoperative knee function in comparison with an anterior cruciate ligament injury. Knee Surg Sports Traumatol Arthrosc.

[13] Bastick AN, Runhaar J, Belo JN, Bierma-Zeinstra SM (2015). Prognostic factors for progression of clinical osteoarthritis of the knee: a systematic review of observational studies. Arthritis Res Ther..

[14] Chen YT, Hou CH, Hou SM, Liu JF. The effects of amphiregulin induced MMP-13 production in human osteoarthritis synovial fibroblast. Mediators Inflamm. 2014:759028. doi:10.1155/2014/759028. Epub 2014 Jul 24. PMID: 25147440. 10.1155/2014/759028 PMC4131469 25147440

[15] Qu H, Li J, Wu LD, Chen WP (2016). Trichostatin A increases the TIMP-1/MMP ratio to protect against osteoarthritis in an animal model of the disease. Mol Med Rep.

[16] van den Berg WB (2011). Osteoarthritis year 2010 in review: pathomechanisms. Osteoarthritis Cartilage.

[17] Lei P, Sun R, Hu Y, Li K, Liao Z (2015). Biomechanic effect of posterior cruciate ligament rupture on lateral meniscus. Int J Clin Exp Med.

[18] Bray RC, Leonard CA, Salo PT (2003). Vascular adaptation of intact joint stabilizing structures in the posterior cruciate ligament deficient rabbit knee. J Orthop Res.

[19] Lei P, Sun R, Li K, Hu Y, Liao Z (2015). Morphological changes and expression of MMPs and TIMPs in rabbit degenerated lateral meniscus after PCL-transection. Int J Clin Exp Med.

[20] Li G, Li K, Zhu Y, Li S, Zhang J (2009). Histological changes of degenerated lateral meniscus after anterior cruciate ligament rupture in rabbits. J Clin Rehabilit Tissue Eng Res..

[21] Narvy SJ, Pearl M, Vrla M, Yi A, Hatch GR (2015). Anatomy of the femoral footprint of the posterior cruciate ligament: a systematic review. Arthroscopy.

[22] Makris CA, Georgoulis AD, Papageorgiou CD, Moebius UG, Soucacos PN (2000). Posterior cruciate ligament architecture: evaluation under microsurgical dissection. Arthroscopy.

[23] Wang J, Ao Y (2004). Study on the articular cartilage degeneration secondary to posterior cruciate ligament rupture in rabbit knee. Chin J Sports Med..

[24] Bluteau G, Conrozier T, Mathieu P, Vignon E, Herbage D, Mallein-Gerin F (2001). Matrix metalloproteinase-1, -3, -13 and aggrecanase-1 and -2 are differentially expressed in experimental osteoarthritis. Biochim Biophys Acta.

[25] Tchetina EV, Squires G, Poole AR (2005). Increased type II collagen degradation and very early focal cartilage degeneration is associated with upregulation of chondrocyte differentiation related genes in early human articular cartilage lesions. J Rheumatol.

[26] Laverty S, Girard CA, Williams JM, Hunziker EB, Pritzker KP (2010). The OARSI histopathology initiative - recommendations for histological assessments of osteoarthritis in the rabbit. Osteoarthritis Cartilage.

